# Repolarization of inflammatory macrophages into reparative stage targeting cannabinoid receptor2: a potential perspective to dampen lung injury/ARDS

**DOI:** 10.3389/fphar.2025.1623857

**Published:** 2025-11-07

**Authors:** Chloe Benedict, Jagdish Chandra Joshi

**Affiliations:** 1 Harrison College of Pharmacy, Auburn University, Auburn, AL, United States; 2 School of Pharmacy, Lake Erie College of Osteopathic Medicine, Erie, PA, United States

**Keywords:** lung injury, ARDS, macrophages, macrophages polarization, cannabinoid agonist, GPR18

## Abstract

The inflammatory response during acute lung injury and ARDS leads to an overactive immune response, causing further damage and irreparable recovery. While there are drugs to target various pathogens that cause acute lung diseases, still, the consequences of infection-induced inflammatory signaling and damage prevention are limited with available drugs. With the rise of cannabinoids as a potential therapeutic agent in several inflammatory disease states, many studies have specifically evaluated their anti-inflammatory effects via CB2 receptors and non-cannabinoid receptors, such as GPR18, in infectious lung injury. However, the exact mechanisms behind CB2 receptor agonism in the application of acute lung injury are still not clear. Lung macrophages are major immune cells that play a major role in checking and defending the primary and secondary consequences of lung infectious injury. The exact mechanism by which macrophages differentiate to produce anti-inflammatory effects over inflammation is still widely debated during episodes of acute lung injury or respiratory distress. Using systematic literature evaluation and analysis of current trends and gaps in the literature, we have analyzed the mechanisms that CB2 agonists involve in dampening inflammatory signaling and redirecting the response in acute lung injuries/ARDS by modifying the nature of inflammatory macrophages to anti-inflammatory. Our systematic review indicated that within the inflammatory macrophage response, CB2 agonists impact several signaling pathways involved in the excessive immune response, reducing the expression of inflammatory transcription factors and inflammatory cytokine storm, and redirecting the macrophages to resolve the lung injury/ARDS.

## Introduction

Acute respiratory distress syndrome (ARDS) is a heterogeneous clinical syndrome that contributes more than 50% mortality in severely ill hospitalized patients and is a major global economic burden (Bardaji-Carrillo et al., 2025). Overactivated pulmonary immune reaction during ARDS releases various inflammatory cytokines, called cytokine storm, and the overload of infiltrated neutrophils in the lungs leads to serious systemic complications (Ma et al., 2025; Joshi et al., 2025). The pneumocytes damage, pulmonary microvascular leakage, neutrophil infiltration, alveolar wall diffusion, edema, and hypoxic respiratory failure are the prominent hallmarks of ARDS following acute lung injury (ALI) (Ma et al., 2025; Rochford et al., 2021; Al-Husinat et al., 2025; Akhter et al., 2021; Rayees et al., 2019). Despite continuous research, more than a half-century of years, for the development of a therapeutic tool (Ragel et al., 2023) there is no specific agent available to treat this devastating syndrome. Pulmonary macrophages are the most important immune cells that plays a vital role in the first line defense against various invading bacterial and viral pathogens, including the SARS-CoV-2 virus (Rayees et al., 2019; Malainou et al., 2023; Rayees et al., 2020). Two primary types of macrophage populations called alveolar and interstitial macrophages reside in the lung. Another population, transcriptionally and phenotypically different, called recruited macrophages, derives from circulatory monocytes during lung injury (Joshi et al., 2020; Misharin et al., 2013). Macrophages are involved in sensing the pathogen and dead cells, thereby internalizing and degrading them by efferocytosis, an important process for resolution of inflammation and maintaining tissue homeostasis (Malainou et al., 2023; Wynn et al., 2013; Gautier et al., 2012; Roquilly et al., 2020). During this process, macrophages orchestrate and resolve inflammation and are generally classified as M1 (pro-inflammatory) and M2 (anti-inflammatory) macrophages. M1 macrophages are marked by the generation of iNOS, TNF-α, IL-6 and IL-12 (Strizova et al., 2023; Dousdampanis et al., 2024; Chen et al., 2023; Lu et al., 2015), while M2 macrophages are marked by the expression and synthesis of Arg1, CD206, and IL-10 (Roszer, 2015; Yu et al., 2025; Luo et al., 2024; O'Brien and Spiller, 2022). Additionally, M2 macrophages are subdivided into M2a, M2b, M2c, and M2d, based on their distinct stimulation, functional and cellular pathway (Roszer, 2015; Wang et al., 2019; Kiseleva et al., 2023). Single cell transcriptomic studies reveal presence of specific markers for various tissue macrophages, such as Clef4 for hepatic macrophage known as Kupffer cells, Tmem119 for microglia (brain macrophages), Siglec-F for alveolar macrophages (Rochford et al., 2021; Flores Molina et al., 2022; Ruan and Elyaman, 2022; Guan et al., 2025). Macrophages that are inclined toward the M1 state display surface makers including MHC II, CD68, CD80, CD86, and CCR2, while macrophages skewed towards the M2 state display the mannose receptor (CD206), CD209, FIZZ1, Ym1/2, TfR, and Dectin-1 (Rochford et al., 2021; Trombetta et al., 2018; Shapouri-Moghaddam et al., 2018; Gordon and Martinez, 2010). In the lung, alveolar macrophages express the CD11c^+^, Siglec-F^+^ phenotypes and are distinguished from recruited monocytes/macrophages with the expression of CD11b^+^ surface marker (Rochford et al., 2021; Joshi et al., 2020). Alveolar macrophages are the first immune cells that encounter invading pathogens, release various inflammatory cytokines, and increase neutrophils influx into the airspace. Macrophages are a heterogeneous group of cells and play a bidirectional role following pathogen insult and clear dead cells debris or pathogen after triggering host defense inflammatory signaling. Based on microenvironmental clues and stimulating factors, these cells change their phenotypes and transcriptomic profiles that render them to perform their M1 vs. M2 functions (Rochford et al., 2021; Rayees et al., 2020; Bain and MacDonald, 2022; Rodriguez-Morales and Franklin, 2023). Therefore, the repolarization of M1 macrophages to M2 stages is required promptly to repair and resolve lung inflammation and cytokine storm during ARDS/ALI.

However, *in vitro,* preclinical and clinical studies suggest CB2 agonist resolves lung inflammation and injury, but the exact mechanism of how these agonists dampen the lung injury is not clear so far. Various studies showed that cannabinoid receptor-2, which is encoded by the Cnr2 gene, is expressed in macrophages and their activation is involved in the resolution of lung infectious injury (Costola-de-Souza et al., 2013; Liu et al., 2020; Nagre et al., 2022). Thus, in this review article, we systematically addressed the expression level of cannabinoid receptors in various lung cells including lung resident macrophages and monocyte-derived recruited macrophages. We showed the evident possible mechanism of cannabinoid receptors involved in transitioning M1 macrophages to M2 phenotypes that make them reparative to resolve ARDS and lung inflammatory injuries.

## Molecular mechanism of macrophages polarization

In most lung disorders, including life-threatening diseases such as acute lung injury, ARDS, and COVID-ARDS, lung macrophages become activated and release inflammatory mediators (Bain and MacDonald, 2022; Grant et al., 2021; Dang et al., 2022; Wendisch et al., 2021; Epelman et al., 2014; Merad and Martin, 2020). Following lung injury, macrophages recruitment via circulating monocytes can be differentiated into M1 or M2 depending upon the cytokine/mediator signaling they encounter. The M1 and M2 nature of macrophages is directed by the surface and/or intracellular receptor signaling, called pathogens/pattern recognition receptors (PRRs). Their nature can be influenced by co-localized immune modulating receptors (Rochford et al., 2021; Chen et al., 2023). Cytokines’ response following inflammation often drives the transition of either M1 or M2 macrophages (Strizova et al., 2023; Chen et al., 2023). The expression of various STAT transcription factors or suppressors of cytokine signaling proteins can also drive M1 vs. M2 expression (Wilson, 2014; Huang et al., 2018).

Sensory receptors/PRRs, such as toll-like receptors (TLRs) family, TLRs 1, 2, 4, 5, and 6 are present on transmembrane surface while a few, TLRs 3, 7, 8, and 9, are cytosolic receptors present in cytosol of macrophages. Similarly, other PRRs, such as nucleotide-binding domain and leucin-rich repeat containing receptors (NLRs), and retinoic acid-inducible gene-1 (RIG-1), AIM2, and cyclic GMP-AMP (cGAS) like receptors (RLRs) are transmembrane and intracellular receptors (Rayees et al., 2020; Dvorkin et al., 2024; Joshi et al., 2022; Kawai et al., 2024; Lavelle et al., 2010; Sundaram et al., 2024; Yoneyama et al., 2024). PRRs regulated the activation and function of macrophages through their downstream adaptor protein signaling such as myeloid differentiated-88 protein and toll-like receptor (TLR) and interleukin receptor (IL-1R) complex TLR/IL-1R, TRIF/TRAM domain etc. (Rayees et al., 2020; Kawai et al., 2024). These receptors sense and recognize the pathogens associated and/or dead cells, called damaged cells associated with molecular pattern proteins, known as PAMP and DAMP, respectively (Rayees et al., 2020; Ma et al., 2024). Following sensation and recognition of PAMP and/or DAMP macrophages get activated, results in release of various proinflammatory cytokines and proteases results in M1 transition (Rayees et al., 2019; Joshi et al., 2020; Ma et al., 2024). Gram negative pathogens such as *Pseudomonas aeruginosa* or the endotoxins, lipopolysaccharides, released by the bacteria or endotoxin products, are the ligand for the TLR4. Their binding to the TLR4 activated downstream signaling which polarizes the macrophages for M1 stage (Joshi et al., 2020; Ouimet et al., 2015; Yang et al., 2025). Similarly, proinflammatory cytokine, IFNγ secretion and its binding at the surface receptor IFNR, on the macrophages make them more inflammatory/M1, classical macrophages (Joshi et al., 2025; Ouimet et al., 2015; Yang et al., 2025; Joshi et al., 2023). Binding of LPS or IFNγ may lead to a metabolic shift, increase in anaerobic glycolysis, increase in the production of iNOS, and reactive oxygen species resulting in sustained inflammation (Joshi et al., 2025; Yang et al., 2025; Joshi et al., 2023). A study showed that LPS reduces miRNA let-7c and induces M1 macrophages polarization (Banerjee et al., 2013). Multiple studies have shown that various miRNAs are involved in the regulation and polarization of M1 macrophages (Khayati et al., 2023). A bioinformatic study by Lu et al. has shown that there are more than 30 genes which are different between M1 to M2 stage. The study has validated some microRNA which are involved in M1 polarized stages such as miRNA-9-5p, miRNA-147, and miRNA-155 (Lu et al., 2016). Similarly, persistent stimulator of type-1 interferon gene (STING) signaling polarizes recruited macrophages towards M1 stage (Joshi et al., 2020; Joshi et al., 2021). Activation of transcription factors NFkB, JAK1/JAK2-STAT1/STAT2 via canonical or non-canonical pathway leads to generation of various inflammatory cytokines resulting conversion of M0 stage to M1 macrophages (Lu et al., 2016; Li et al., 2020; Mahjoor et al., 2023).

In the lung, alveolar and/or interstitial and/or recruited macrophages are influenced or differentiated under the impact of granulocyte macrophages colony stimulating factor (GM-CSF) or macrophages colony stimulating factor (M-CSF) (Rumore-Maton et al., 2008). These stimulating factors lead to differentiation of their phenotypic and transcriptomic profile following lung infectious inflammatory injury and resolution period (Rumore-Maton et al., 2008; Bhattacharya et al., 2015). Increased level of GM-CSF during inflammatory injury mimics the conversion of monocytes to M1 macrophages (Rayees et al., 2020; Joshi et al., 2020). Binding of GM-CSF to its receptor leads to JAK2/STAT5 signaling, induction of NFkB, IRF5, cytokines such as IL-6, IL1β, TNFα, and M-CSF production (Rumore-Maton et al., 2008; Bhattacharya et al., 2015).

Macrophages express various phagocytic receptors such as TIM and TAM family members for engulfing the dead cells and pathogens, a hallmark for M2 macrophages (Moon et al., 2023; Zheng et al., 2023). Activation of Rac1, a small GTPase family signaling protein, in M2 macrophages increase the apoptosis of phagocytized dead cells and pathogens (Moon et al., 2023; Zheng et al., 2023). However, there are various controversial reports for macrophages convergence towards M1 or M2 stage by protease activated receptor2 (PAR2) activation, a Gq protein linked GPCR (Rayees et al., 2019; Rayees et al., 2020). A recent study by Joshi et al. has shown that the RGS2 protein linked with Gq protein signaling regulates the IFNγ induced production of M1 macrophages and transits them to M2 stage (Joshi et al., 2025; Joshi et al., 2023). Similarly, studies have shown that the second messenger, cAMP, signaling suppresses calcium dependent transcription factor, NFAT, and inflammatory cytokines to repolarize macrophages to M2 stage (Rochford et al., 2021; Rayees et al., 2019). Th2-cytokines, anti-inflammatory in nature, such as IL4/IL13, TGF-β and IL-10 polarizes macrophages towards M2 stage though activation of STAT3 and STAT6 transcription factor (Rayees et al., 2020; Guan et al., 2025). Activation of STAT6 through IL4 signaling is also involved in the suppression of STAT1 downstream inflammatory signaling (Rayees et al., 2020). Similarly, peroxisome proliferator-activated receptor-γ (PPARγ), a member of nuclear receptor family, is a regulator of macrophage polarization by making a metabolic shift and generation of IL4 and IL13 (Yao et al., 2018).

## Expression of cannabinoid receptors in lung macrophages

**FIGURE 1:**
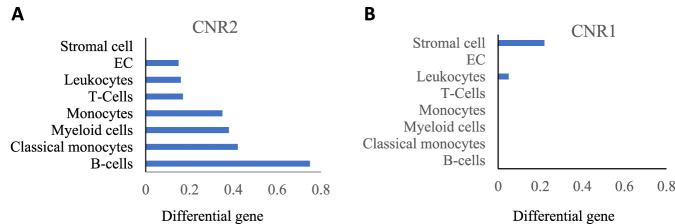
**(A)** CNR2 (CB2 receptor) expression; **(B)** CNR1 (CB1 receptor) expression in indicated lung cells acquired from Tabula Muris data.

Two types of well-known cannabinoid receptors, cannabinoid1 (CB1) and cannabinoid2 (CB2), which were cloned in 1990 and 1993, respectively, are ubiquitously present in mammals and share 44% similar amino acids (Munro et al., 1993; Becker-Baldus et al., 2023). Both CB1 and CB2 are G-protein coupled receptors (GPCRs) belonging to the rhodopsin family, with CB1 being a 473 and CB2 a 360 amino acid long chain, respectively. Their activation by the cannabinoids leads to various downstream signaling, depending upon the G-protein coupled with them (Bow and Rimoldi, 2016; Cabral and Griffin-Thomas, 2009). In the lungs, cannabinoid receptor distribution differs in various structural cells and immune cells. Existing literature suggests that the cannabinoid component and its receptors are well expressed in lung macrophages and other immune cells (Gertsch, 2016). The presence of these receptors in macrophages impacts lung immunity and homeostasis mechanisms. We assessed the publicly available data and found that, in the lung, monocytes and myeloid cells, precursors of recruited as well as tissue resident macrophages population, expressed the CB2 receptors (encoded by CNR2 gene) (Figure 1A) (Tabula Muris Consortium, 2018). While the CB1 receptors (encoded by the CNR1 gene) are almost absent in these cells (Figure 1B). Studies suggest that CB1 receptors are mainly expressed in the neuronal system and less in the peripheral system while CB2 receptors are mainly expressed in peripheral immune cells such as macrophages and T-cells, that regulate innate and adaptive immunity (Moe et al., 2024). The publicly available data and literature suggest the higher expression of CB2 receptors in lung macrophages (Munro et al., 1993; Turcotte et al., 2016), that might be responsible for the modulation of lung inflammation. Thus, this manuscript focuses on the depth of CB2 receptor signaling, and its effect on lung homeostasis and resolution.

## Crosstalk of CB2 and GPR18 downstream signaling in macrophages

**FIGURE 2:**
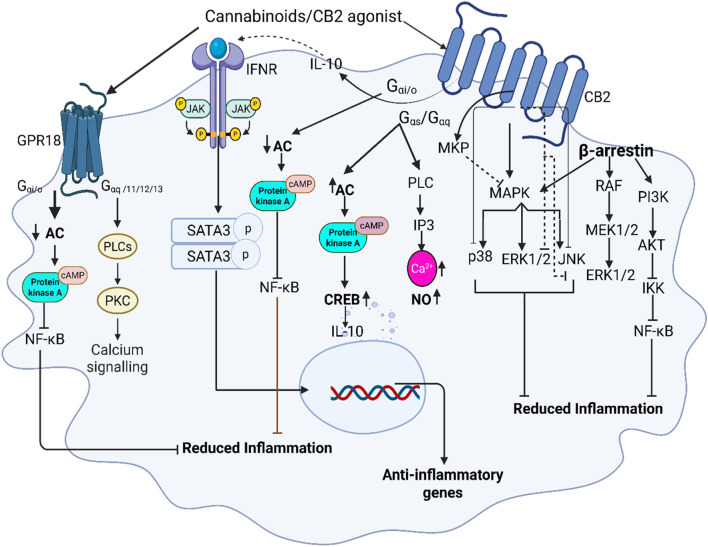
Illustrates the role of cannabinoid receptor signaling in macrophages, emphasizing the interaction between CB2 and GPR18 receptors. Upon binding to CB2 receptors, CB2 agonists activate Gi/o protein-coupled signaling, leading to the inhibition of adenylate cyclase and subsequent reduction in cyclic AMP (cAMP) levels. cAMP inhibits the activation of protein kinase A (PKA) and nuclear factor kappa B (NF-κB), suppressing pro-inflammatory cytokine production while promoting anti-inflammatory macrophage polarization via generating IL-10-JAK/STAT3 signaling and inhibiting MAPK directly by inducing mitogen-activated protein kinase-phosphatases (MKP) as well as by inhibiting its downstream pathways. Recruitment of β-arrestin initiates RAF/MEK/ERK signaling, however, its final ERK pathway is inhibited by CB2, additionally β-arrestin inhibits NF-κB through PI3K/AKT signaling. GPR18, co-expressed with CB2 in macrophages, activation leads to inhibition of NF-κB via PKA and increased intracellular calcium mobilization further contributing to anti-inflammatory responses.

CB2 receptors are coupled with G_i/o_ protein and their activation results in the inhibition of adenyl cyclase which inactivates protein kinases-A and inhibits the activation of transcript factor NF-κB while modulating mitogen-activated protein kinase (MAPK) (Bow and Rimoldi, 2016; Cabral and Griffin-Thomas, 2009; Fernandez-Ruiz et al., 2007) (Figure 2). The interesting nature of CB2 activation is seen by the initial suppression of cAMP and later increased expression of cAMP levels (up to 10 folds) in T-cells, thereby inhibiting the T-cells’ signaling and performing immunosuppressive effects (Bow and Rimoldi, 2016). Studies show that a selective CB2 agonist leads to the inhibition of p27Kip1, a cyclin-dependent kinase inhibitor, and activates phosphatidylinositol 3-kinas3 (PI3K)/serine-threonine kinase (Akt)/mammalian target of rapamycin complex 1 (mTORC1). Activation of PI3K/AKT/mTORC1 signaling pathway, results in proliferation of neuronal progenitor cells and breast cancer (Palazuelos et al., 2012; Song et al., 2023). Similarly, studies by Choi et al. have shown that CB2 agonism activates adenosine monophosphate-activated protein kinase (AMPK) and cyclic adenosine monophosphate response element binding (CREB) protein, thereby reducing cerebral ischemia (Choi et al., 2013). Additionally, CB2 receptors are partly associated with Gαs and Gαq proteins in different cells and their signaling varies depending on the nature of cells and the presented receptor type (Brust et al., 2023; Saroz et al., 2019). Therefore, Gαs stimulation by the CB2 receptor might be responsible for the modulation of cAMP levels (Saroz et al., 2019; Mensching et al., 2020) in T-cells (Bow and Rimoldi, 2016). Furthermore, coupling with Gαq proteins leads to the activation of phospholipase C and inositol 1,4,5-triphosphate (IP3) signaling and thereby increases intracellular calcium levels (Cabral and Griffin-Thomas, 2009).

Several studies have examined the impact that CB2 agonists have on transcription factor NF-κB and its precursors which is upregulated by the phosphorylation of IkB (Solt and May, 2008). Liu et al., indicated that treatment with CB2 agonists, JWH133, results in inhibition of IkB degradation and phosphorylation thereby dampening the effect on NF-κB activation and promoting anti-inflammatory responses in the lungs (Liu et al., 2014). According to Mormina et al., this effect also leads to the regulation of the pro-inflammatory cytokine IL-8 release in colon cells (Mormina et al., 2006).

CB2 agonists have been shown to increase IL-10 response in both microglia and macrophages in eyes, leading to the activation of STAT3 (Olabiyi et al., 2023; Nakamura et al., 2015). STAT 3 signaling is responsible for dampening cytokine storms seen in inflammatory disease states (Hu et al., 2014; Hutchins et al., 2013). By enhancing the biosynthesis of IL-10, CB2 receptors indirectly trigger the signaling cascade of the JAK/STAT3 pathway in macrophages which decreases key inflammatory cytokines such as TNF-α and IL-6 (Hu et al., 2014). Activation of the above signaling cascades may also play a significant role in the regulation of Th1 or Th2 signals which are responsible for macrophage repolarization (Correa et al., 2005).

**FIGURE 3:**
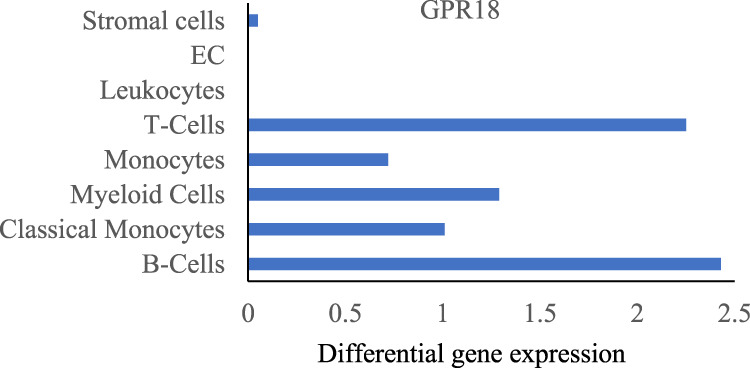
GPR18 expression in indicated lung cells acquired from Tabula Muris data.

Non-cannabinoid receptors such as GPR18 and GPR55 are known as the targets of cannabinoids. Among them GPR18, coupled with Gαi/o, Gαq/11, Gα12/13, and β-arrestin proteins, is highly expressed in lung macrophages and stimulated by CB2 agonist, (Figure 3) (Tabula Muris Consortium, 2018), is involved in the management of inflammation (Laprairie et al., 2017; Morales and Reggio, 2017).

Several studies have shown that a range of endogenous, phytogenic, and synthetic cannabinoids can modulate GPR18 (Morales et al., 2020). The study also suggested that there is a functional heteroreceptor interaction and energy transfer between CB2 receptors and GPR18 (Reyes-Resina et al., 2018). *N*-arachidonoyl glycine (NAGly), resolvin D2, *N*-arachidonoyl ethanolamine (AEA), Abn-CBD, and Delta-9 THC are a few of the ligands that bind to GPR18 and have the potential to produce anti-inflammatory effects in the lungs as seen in CB2 activation (Morales et al., 2020). While the specific ligand resolvin D2 does not directly bind with the CB2 receptor, a study performed on murine lung PMN cells indicated that once bound, resolvin D2 leads to a protective effect in I/R lung injury by lowering PMN infiltration (Morales et al., 2020; Chiang et al., 2015).

GPR18 has been shown to signal through both Gαi/o and Gαq/11 (Morales et al., 2020). Findings by Console-Bram et al. indicated that GPR18 activation inhibits PKC (Console-Bram et al., 2014). A study by Fabisiak group has shown that GPR18 agonists have potent anti-inflammatory role in intestinal inflammatory disease and reduced TNF-α, IL-6 and myeloperoxidase activity (Fabisiak et al., 2021). Co-expression of GPR18 and CB2 receptors in macrophages and coupling with similar G-protein clearly shows the crosstalk between these receptors and their synergic effects. Studies have shown that CB2 receptor activation inhibits the downstream effects of MAPK by attenuating ERK 1/2, JNK, and p38 signaling to resolve lung injury. Selective CB2 agonist, JWH133, inhibited MAPK, and NF-κB activation, leading to reduction of various inflammatory cytokines (Liu et al., 2014; Hashiesh et al., 2021). Furthermore, a study in LPS-exposed microglial cells have shown that CB2 activation results in anti-inflammatory cell phenotypes through the induction of mitogen-activated protein kinase-phosphatases (MKP), MKP-1 and MKP-3, a negative regulator of MAPK and subsequent dephosphorylation of ERK and p38 leading to the reduction of inflammatory mediators (Romero-Sandoval et al., 2009; Chen et al., 2021).

Moreover, Recchiuti et al. determined that resolvin D2 decreased the release of IL-8, TNF-α, and NF-κB, all key players of the cytokine storm, and may also play a role in macrophage differentiation to the anti-inflammatory state (Recchiuti et al., 2021). In addition to this, nonselective cannabinoid agonists, AEA and delta-9-tetrahydrocannabinol have been studied as agonists that impact both CB2 and GPR18 (Morales et al., 2020). One such study revealed that anandamide can induce efferocytosis in human primary monocyte-derived macrophages (MoDMs) by engaging both CB2 and GPR18 (Leuti et al., 2024). This process is crucial in resolving inflammation and contributes to the production of pro-resolving lipids. GPR18 signaling is also involved in engaging the MAP kinase pathway and calcium mechanisms through the binding of NAGLy, delta-9 THC, and Abn-CBD (Console-Bram et al., 2014). Determining such mechanisms will allow for a greater understanding of GPR18 as a cannabinoid receptor and its potential use in dampening inflammatory pathways in the lungs.

The role of G proteins after CB2 receptor activation in acute lung injury is associated with anti-inflammatory effects and differentiates from the recruitment of β-arrestin (Ibsen et al., 2019). The CB2 agonism impact in β-arrestin role is controversial. Although, a few studies suggested β-arrestin leads to the internalization of CB2 receptors that trigger the release of pro-inflammatory signaling (Turu et al., 2021). A recent study has shown that selective CB2 agonists induce the recruitment of β-arrestin and inhibit adenylyl cyclase (Valeriano et al., 2025). Feng et al., has shown that β-arrestin regulates pro-inflammatory cytokine production by interacting with transforming growth factor-β-kinase1 (TAK1)-binding protein1 (TAB1) in endotoxin induced-microglia. Interaction of β-arrestin with TAK1-TAB1 reduces expression of pro-inflammatory gene and cytokine production (Feng et al., 2014). In addition, CB2 agonists can bind β-arrestin1 and β-arrestin 2, leading to receptor internalization and the potential phosphorylation of MAPK, ERK 1/2 (Rakotoarive et al., 2024). In conditions, such as exposure time to CB2 agonist and functional selectivity, β-arrestin activation can lead to downstream effects of ERK 1/2 signaling and can also form a scaffolding complex with Raf-a, MEK, and ERK 1/2 (Dhopeshwarkar and Mackie, 2014). A study by Dhopeshwarkar and Mackie in HEK cells has shown minimal efficacy of CB2 agonist for β-arrestin2 (Dhopeshwarkar and Mackie, 2014). A systematic study by Ibsen et al. suggests that there is a differential effect of CB2 in the translocation of β-arrestin1,2, in human vs. bovine (Ibsen et al., 2019).

## Regulation of macrophages polarization by CB2 receptor signaling

**FIGURE 4:**
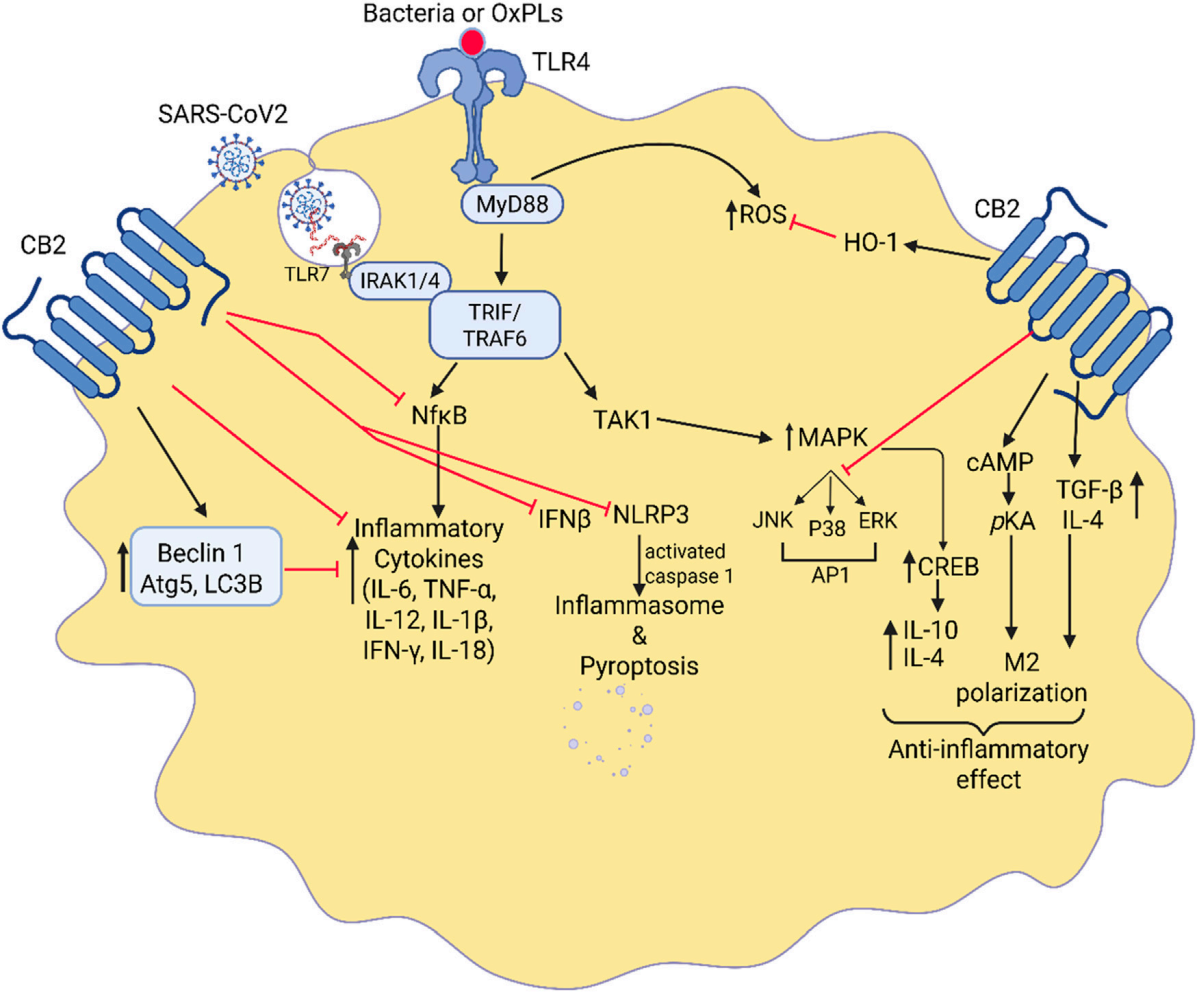
Interaction between CB2 signaling and TLR-4-induced immune response. Highlights of the crosstalk between CB2 agonists and TLR-4 signaling in the regulation of lung inflammation. It illustrates how CB2 receptor activation counteracts TLR-4-induced immune responses, which are key drivers of acute lung injury (ALI) and ARDS. Upon activation, CB2 receptors inhibit TLR-4-mediated pathways, suppressing the NF-κB and MAPK signaling cascades. This results in a reduction of pro-inflammatory cytokines such as IL-1β, TNF-α, and IL-18 while promoting anti-inflammatory mediators like IL-10. Additionally, CB2 agonists modulate macrophage polarization, shifting them from a pro-inflammatory (M1) to an anti-inflammatory (M2) state. This interplay ultimately dampens excessive immune activation, prevents neutrophil infiltration, and promotes lung tissue repair.

Cannabinoids and CB2 receptors are expressed in various peripheral immune cells, but their expression in macrophages is increased during inflammation (Lin et al., 2022). CB2 receptors’ downstream signaling regulates various inflammatory signaling by suppressing immune-mediated inflammation or by interacting with transcription factors that regulate the inflammation (Figure 4). CB2 receptors activated in macrophages suppress TLR4-induced immune signaling (Cui et al., 2024). It has been shown that CB2 receptor activation greatly affects acute disease states by regulating the response of key immune cells such as macrophages during chronic autoimmune inflammatory processes (Rakotoarive et al., 2024; Capozzi et al., 2021). Cannabinoids have also been shown to produce anti-inflammatory actions by inhibiting the specific proteins and pathways that arise from TLR-4 activation. For instance, inhibition of the NLRP3 inflammasome, which is induced in septic lung injury (Liu et al., 2020; Cui et al., 2024). By using RAW 264.7 macrophage cell line, Liu et al. confirmed that in the presence of a CB2 agonist several autophagic genes such as Beclin-1, Atg5, and LC3B were increased (Liu et al., 2020). Upregulation of autophagy genes inhibits IL-12, IL-18, TNF-α, IL-1β, and NLRP3 inflammasomes, leading to reduced excessive inflammatory response and cell death found in septic lung injury (Liu et al., 2020; Capozzi et al., 2021). Similarly, CB2 activation in human immunocompetent cells stimulates TGF-β and IL-4 while inhibiting IL-12, IL-17, TNF-α, IFNγ (Saroz et al., 2019). A study performed on murine peritoneal macrophages demonstrated that CB2 activation amplifies the anti-inflammatory cytokine IL-10 and downregulates the inflammatory response (Tomar et al., 2015). Another study performed on human peripheral blood mononucleated cells in celiac disease subjects demonstrated that CB2 receptor activation amplified the anti-inflammatory cytokine IL-10 (Tortora et al., 2022) and thereby downregulates the inflammatory response in macrophages. Tahamtan et al., demonstrated the anti-inflammatory effects of CB2 agonists in respiratory syncytial virus (RSV) in human and animal and indicated that CB2 activation enhanced IL-10 production and reduced bronchoalveolar cellular influx and IFN-γ and MIP-1α production leading to control of RSV disease (Tahamtan et al., 2018). IL-10 acts as a negative feedback mechanism to prevent any tissue damage that would occur from normal inflammatory processes. Similarly, CB2 activation-induced MAPK enhances the phosphorylation of cyclic AMP response element-binding (CREB) protein and results in the generation of IL-10 (Choi et al., 2013). The study by Saroj et al. has also shown that the generation of IL-10 in leukocytes could be due to the activation of Gαs protein of GPCR by CB2 ligands (Saroz et al., 2019). Studies have also shown that the use of CB2 agonists prevented the thrombin-induced polarization of the M1 phenotype cells in microglia while promoting M2 polarization by activation of the cAMP/PKA pathway and anti-inflammatory cytokines, IL-4, IL-10, including BDNF (Tao et al., 2016; Tanaka et al., 2020). Likewise, the CB2 agonist JT11 was found to modulate the MAPK, ERK1/2, and NF-kB-p65 inflammatory transcription factors and lead to the downregulation of pro-inflammatory signaling in PBMCs from healthy donors (Capozzi et al., 2021). Furthermore, CB2 receptor activation inhibits the TLR4-induced p38 MAPK pathway and redirects the inflammatory response in neuroinflammatory diseases (Borgonetti et al., 2022). The study by Cho et al. has shown that proinflammatory cytokines produced by macrophages following LPS and D-galactosamine induced fulminant liver injury was inhibited by β-caryophyllene, a Korean bioactive herbal ingredient, selective CB2 agonist (Cho et al., 2015). Zhang et al. showed that activation of the CB2 receptors on lung tissue of CLP-induced sepsis blocked the inflammatory cytokines IL-18 and IL-1β and decreased pyroptosis associated with lung damage in murine bone-marrow-derived macrophages (Zhang et al., 2021). A recent study by Valeriano group has shown that CB2 agonist, GP1a, reduced the production of TNF-α, IL-6, iNOS, COX-2 and lipid droplets, hallmark of M1 macrophages, in *Bacillus* Calmett-Guerin (BCG) stimulated murine macrophages by modulating metabolic programming and transcription factors (Valeriano et al., 2025).

The CB2 receptor activation has also been shown to have anti-inflammatory and hepatoprotective effects during alcoholic liver disease through an autophagy-dependent pathway in Kupffer cells (Tomar et al., 2015). Denaes et al. demonstrated that the anti-inflammatory, autophagic effects of CB2 activation on liver damage are mediated through the induction of HO-1 which has been shown to play a significant role in dampening the pro-inflammatory response to LPS in Kupffer cells (Denaes et al., 2016). A study by Du et al. suggested that CB2 activation reduced infiltrations of inflammatory macrophages and increased the proliferation of anti-inflammatory macrophages during wound healing (Du et al., 2018). Similarly, a study by Liu et al., in zebrafish has shown that activation of CB2 receptors inhibits the diapedesis of inflammatory cells by modulating the JNK-Alox5 pathway (Liu et al., 2013).

Studies showed that following LPS induces ALI/ARDS, CB2 agonist, HU308, reduced systemic inflammatory cytokine and chemokines release, adhesion of polymorphonuclear cells to the pulmonary capillary, and infiltration of neutrophils in the alveolar space (Hall et al., 2022). The study in primary human monocytes and brain microvascular endothelial cells has shown that CB2 activation reduced the formation of lamellipodia, integrins, and lymphocyte function-associated antigens in monocytes. Thus, this study confirms that the factors that are important for monocyte/macrophage migration and activation are suppressed by CB2 agonism in these cells and thereby inhibit inflammation (Rom et al., 2013). Studies have found that the increased expression of CB2 receptors in microglia in AD and HD patients counter the inflammation and toxic effects by suppressing NO-induced free radical generation and inflammatory cytokines (Guo et al., 2022). Thus, suppression of proinflammatory cytokines and NO generations in microglia, macrophages in the brain, is a critical step to converting microglia from inflammatory to reparative stages (Guo et al., 2022; Morcuende et al., 2022). It is also observed that CB2 receptor activation enhances the IL1R antagonist effect and suppresses inflammation (Fernandez-Ruiz et al., 2007). Thus, more preclinical and clinical studies are needed to be conscious of the effect of CB2 activation on lung inflammation and recognize the repolarization mechanism of inflammatory macrophages to resolve lung injury.

## Limitations of CB2 agonist preclinical studies in lung injury/ARDS model

Preclinical pharmacodynamic and pharmacokinetic studies of CB2 agonists are a necessary step in developing clinical studies. While there have been several preclinical trials that imply the utility of CB2 agonists in treating inflammatory lung disease, fibrosis, neurodegeneration, and cancer, the translation of those outcomes into humans remains difficult to elicit due to the differences in the physiological and pathological processes between the two models (Whiting et al., 2022; Carruthers and Grimsey, 2024). One of the difficulties in applying animal data to humans is due to the large degree of primary amino acid sequence divergence between humans and rodents including divergence in the c-terminal and n-terminal tail of the CB2 receptor which are largely responsible for β-arrestin engagement and intracellular trafficking (Whiting et al., 2022; Carruthers and Grimsey, 2024; Howlett and Abood, 2017). Additionally, small animals such as mice and rats are among the primary test subjects of CB2 agonists and tend to have different ligand engagement and signaling responses compared with humans (Carruthers and Grimsey, 2024; Li et al., 2023; Zhang et al., 2015; Li et al., 2019). There is also some discrepancy between previous clinical trials and preclinical data. Recent preclinical data strongly supports the potential efficacy of CB2 agonists in inflammatory and fibrotic diseases. Past clinical data on these compounds focused primarily on conditions related to pain, failing to truly reflect the results found in preclinical trials. The mismatch in patient populations between preclinical and clinical trials makes it challenging to fully apply the results of animal models to humans (Atwood et al., 2012). And so, clinical trials that have a strong focus on CB2 agonists’ role in inflammatory and fibrotic diseases may be more likely to have successful outcomes in practice. These specific differences in the polypeptide components of CB2 receptors and signaling responses in preclinical models must be considered when developing clinical drug candidates (Soethoudt et al., 2017; Wu et al., 2022).

In several preclinical models, JWH133, a selective CB2 receptor agonist, has been extensively studied to determine its safety and efficacy in various lung diseases. As mentioned previously, Lie et al. found that JWH 133 reduced paraquat induced lung edema and pathology. While the overall study design included proper time intervals, control groups, and biological efficacy markers, it did not include CB2 selective antagonists or knockout animals and could not necessarily be applied to situations outside of paraquat poisoning (Liu et al., 2014; Hashiesh et al., 2021)}. These characteristics of preclinical study would provide more information on the applicability of JWH-133 in lung injury. Furthermore, a study performed in mice investigated the impact of JWH133 on nicotine induced lung fibrosis. The results of this study were promising in demonstrating CB2R agonist protective effects in lung fibrosis. However, the study did not fully address any safety biomarkers or dose equivalence in humans (Wawryk-Gawda et al., 2018). Thus, the complete pharmacokinetic profile including acute, subacute, and chronic toxicity studies are required to translate into clinical trials which are lacking so far (Hashiesh et al., 2021). Recently, a study by Nicholson et al. using a mice model for sulfur mustard toxicity induced ALI examined the effect of CB2 agonist, HU308. The results indicated that HU308 significantly reduced pro-inflammatory cytokines and immune cell infiltration, demonstrating HU308 as a therapeutic agent for pulmonary injury (Nicholson et al., 2025). The translatability of this study into human clinical trials is still questionable though as the evaluators only used an analog of sulfur mustard which may not fully recapitulate clinical lung damage and ARDS models. Additionally, HU308 was administered intraperitoneally which could not be a feasible and specific route of drug administration for ARDS patients when translating from bench to bedside (Al Shoyaib et al., 2019).

Ultimately, there is a greater need for modified animal models which can mimic clinical ARDS and signaling cascades in humans (Carruthers and Grimsey, 2024). As technology develops, PET and fluorescent ligands may also be helpful in clarifying our understanding of *in vivo* CB2 expression and drug distribution (Whiting et al., 2022; Bhattacharjee et al., 2023). Creating CB2R agonist therapies is further challenging due to the complexity of the lipophilic nature of many natural and synthetic agonists. Endogenous agonists of class A GPCRs tend to be hydrophilic which is opposite of endocannabinoids lipophilic nature. This suggests that CB2 agonist binding and signaling may affect newly synthesized drugs or natural cannabinoids’ pharmacodynamics (Li et al., 2023). Translational success may also depend on overcoming the risk for immune suppression upon chronic use or pro-inflammatory actions (Navarro et al., 2016; Zhou et al., 2016).

## Clinical relevance and challenges of CB2 agonist’s preclinical findings

Clinical trials involving CB2 agonists are lacking and have not been successful in producing safe outcomes for marketing those products to use in clinical settings. A handful of clinical trials for CB2R agonists, especially in relation to acute lung injury and ARDS, are underway and have shown promising results. *Lenabasum*, also referred to as *alujemic acid*, is the primary CB2 agonist that has been tested in lung-related diseases in humans. Trials have indicated its ability to decrease inflammation associated with cystic fibrosis. Chmiel et al. showed in a randomized, placebo-controlled phase 2 trial that there was a reduction in inflammatory cells and mediators in the sputum of patients who used *lenabasum* for 12 weeks. Potential concerns of this trial included small sample sizes, shorter treatment duration, and lack of strong efficacy data, all of which must be determined to approve its application (Chmiel et al., 2021). In contrast, West el al. more recently demonstrated that *lenabasum* did not improve clinical outcomes such as pulmonary exacerbations in a single-center, double-blind, randomized, placebo-controlled phase 2b study (West et al., 2025). Another clinical trial tested *lenabasum’s* safety and efficacy profile in patients with dermatomyositis. *Lenabasum* treatment was well tolerated and led to a greater improvement in dermatomyositis inflammatory markers (Werth et al., 2022). Despite promising therapeutic potential offered by CB2R agonists, their translational success in humans depends on overcoming certain limitations as no CB2 receptor agonists have been approved as therapeutic candidates (Meanti et al., 2025). In the future, larger studies with placebo control and greater randomization would provide more helpful clinical data in humans.

Even though there is limited clinical data available, CB2R agonists have also been shown to play a significant role in mitigating the damage in extrapulmonary systems such as neurodegenerative inflammatory disease. One systematic review using more than 565 papers on Parkinson’s disease, only found 7 randomized trials that examined the effect of cannabinoids treating the motor signs of PD. These results from the trials did not support the efficacy in treating these patients due to a lack of evidence (Meanti et al., 2025; Basil et al., 2022). Moreover, there is another potential for novel CB2R agonist NTRX-07 to be incorporated into a clinical trial that focuses on treating glioblastoma tumors. NTRX-07 is an oral CB2 agonist that was shown to exert potent anti-cancer activity when administered at a dose of 300 mg/kg (Kiraly et al., 2023; Feng et al., 2024). Determining the most effective dose with radiation therapy would be beneficial in utilizing NTRX-07 even more so in clinical scenarios. One other pilot clinical trial investigated the effects of Sativex, an oromucosal spray with THC and CBD in Huntington’s Disease patients. This was a randomized, double bind study that administered up to 12 sprays per day over 12 weeks {Lopez-Sendon Moreno, 2016 #187). The study aimed to assess the safety and efficacy of ECS modulation in patients but requires additional research to establish definitive therapeutic outcomes. Clinical studies, although limited, suggest that CB2 receptor activation may offer neuroprotection and symptomatic relief, but new well-designed trials are needed (Meanti et al., 2025).

Two of the earliest-evaluated CB2 selective agonists, PRS-211,375 and GW842166, were also investigated in clinical trials for the treatment of pain and are 300-fold selective for CB2 over CB1 (Whiting et al., 2022). In Phase 2a trials, PRS-211,375 demonstrated efficacy in patients with a third molar extraction with the lowest administered dose, but not at higher doses. It ended up failing in a Phase IIb study as it did not show efficacy in reducing pain (Bow and Rimoldi, 2016; Whiting et al., 2022). In addition, GW842166 was well tolerated but demonstrated lower efficacy in Phase 2 trials for osteoarthritis and dental pain (Ostenfeld et al., 2011).

More recently, there has been a new CB2R agonist that will be utilized in a clinical trial. RaQualia pharma is initiating a phase 1 clinical trial of, RG-00202730, to determine its clinical impact for chemotherapy induced peripheral neuropathy. Even though this is not related to the inflammatory process of lung disease, the study will provide more information on this potential drug class as a whole and give insight on pharmacokinetic and pharmacodynamic effects of a CB2 agonist in humans (RaQualia Pharma Inc, 2023).

## Potential safety implications for CB2 agonists in clinical and preclinical trials

CB 2 agonists have been shown to be primarily expressed in the peripheral tissues of the body including the spleen, bone, heart, and adipose tissue (Yang et al., 2012; Bie et al., 2018). While initial studies indicated CB2 receptors were not present in the CNS under normal physiological conditions, more recent studies indicate that CB2 receptors do exist within the central nervous system (Navarro et al., 2016; Grabon et al., 2023). Concerns for off-target effects such as cognitive impairment are a key issue when it comes to developing therapeutics with CB2 agonists for non-CNS disease states (Roche and Finn, 2010). Selectivity of compounds for CB1 vs. CB2 receptors is crucial in determining the pharmacodynamic effects of cannabinoids in humans (Han et al., 2013). Compounds with greater selectivity for CB2 receptors may lead to lesser effects on the CNS as these receptors are profoundly present in peripheral tissue (Sholler et al., 2020). Preclinical and clinical data have suggested the safety profiles of Cb2 agonists in various disease states. The clinical study with *lenasabum* proposed its safe use in patients. At the end of the treatment duration, there were no reports of serious adverse events or intolerability issues. There was a 4.9% study discontinuation rate due to treatment-related adverse events. Overall, *lenasabum* could advance to a larger trial because of positive safety outcomes. APD 371 is a CB2R agonist that has the potential to modulate the pain response. In one clinical trial, the study results confirmed that there were no psychotropic side effects seen or clinically significant changes in vital signs when utilizing APD 371 (Jones et al., 2018). In addition, an open label phase 2a study on *olorinab*, a peripherally acting CB2 agonist, examined its safety/tolerability, pharmacokinetics and efficacy profiles in patients with Crohn’s disease with abdominal pain. This study included 14 subjects who were given *olironab* for 8 weeks. It demonstrated mild to moderate adverse effects and higher efficacy in reducing pain (Yacyshyn et al., 2021; Chang et al., 2023). Their findings suggested 30 milds to moderate treatment emergent adverse events none of which required treatment discontinuation, dose reductions, or dose interruptions. There were also no CNS side effects documented (Li et al., 2023; Yacyshyn et al., 2021). This study confirmed that *olorinab* has 1,000 times higher Cb2 receptor selectivity compared to Cb1 which reduces its ability to have off target activity/side effects (Yacyshyn et al., 2021). More trials with high selective CB2 agents would reduce the concern for harmful safety profiles while using them for peripheral inflammatory disease.

## Future prospective of CB2 agonist in pulmonary diseases

The anti-inflammatory effects of cannabinoids have demonstrated an ability to attenuate the acute and chronic conditions that lead to significant health issues in compromised patients (Peltner et al., 2023; Kalbfell et al., 2023; Nagarkatti et al., 2009). While the clinical data for CB2 mediated anti-inflammatory response in lung inflammation is limited, several animal studies and preclinical data suggest that CB2 mediated effects in inflammatory processes are applicable for multiple etiologies of ARDS including COVID-19 and bacterial pneumonia. The future implications of CB2 receptor agonists and their role in treating pulmonary diseases involve several mechanisms that may dampen the processes that create the inflammatory signals, especially in acute lung injury and inflammation. So far, studies have demonstrated the impact of CB2 agonists on the downregulation of an overactive immune system and their anti-inflammatory actions. There is a need for more data on the direct and safe use of CB2 agonists so that they can be therapeutically utilized for immune-mediated reactions and acute inflammation. CB2 receptors continue to be a potential target for reducing the inflammation involved in lung diseases such as pulmonary fibrosis, lung ischemia, sepsis-associated acute lung injury, and non-small cell lung cancer growth (Liu et al., 2020; Wawryk-Gawda et al., 2018; Wu et al., 2023; Huang et al., 2020; Zeng et al., 2019; Khan et al., 2018). The anti-inflammatory effects of CB2 receptor agonists could lead to it being used as a new therapeutic option in otherwise drug-resistant diseases as seen in its protection against *Pseudomonas aeruginosa*-induced acute lung injury and inflammation (Nagre et al., 2022). Along with this, there has been research to suggest that CB2 receptor agonists may be a therapeutic target for SARS-CoV-2 in targeting viral-mediated immune inflammatory pathogenesis (Nagoor Meeran et al., 2021). Overall, CB2 agonists are utilized to dampen the pathogen specific macrophage responses in ARDS across various etiologies. However, its impact is likely pathogen insult depending on modulating unique transcriptional programs activated by viral vs. bacterial pathogens. ARDS is a heterogeneous syndrome, and macrophage responses are niche depended on, thus, further studies are warranted to establish whether CB2 agonists signaling are broadly effective or it is a more selective intervention.

More research in this area could be promising for alleviating the effects of COVID-19 and similar respiratory illnesses. CB2 receptors may even be one of the more favorable drugs in treating such diseases, since they are involved in nociception, acting at MAP38 kinase inhibitors and reduction of inflammatory status (Aghazadeh Tabrizi et al., 2016). There is room for additional research to be done on the impact of CB2 agonists on pneumonia-induced acute lung injury as potent agents have been shown to improve the clinical outcome of patients with this disease (Hall et al., 2022). The impact that CB2 agonists have on modulating the immune cell function points to its potential to act as a treatment option for asthma or COPD (Parlar et al., 2021; Ferrini et al., 2017). More clinical trials that examine safety and efficacy need to be performed to confirm their future implications and use. Additional pre-clinical and clinical trials that look at the pharmacodynamic effects of CB2 receptor agonists may be useful in determining its true therapeutic effects, as not all previous trials have proven successful. Creating trials with large sample sizes, proper ethical considerations, correct routes of administration, and adequate follow-up time would promote successful outcomes. Comparing CB2 agonists to standard care treatment options for lung disease would provide a broader scope of use for these agents. In addition, discovering the right dosage and route of administration for CB2 agonists in pulmonary disease will be necessary in determining the appropriate pharmacokinetics of this endogenous ligand. The CB2 receptor is highly useful as it does not induce the psychoactivity associated with CB1 receptor activation. Therefore, products that stimulate CB2 receptors need to have high affinity and selectivity for CB2 to avoid adverse off-target effects. Studies that focus on enhancing the physico-chemical aspect of CBD products and their target could show potential in producing the wanted therapeutic effects (Rakotoarive et al., 2024). The information provided in this article displays a comprehensive look into the different pathways that could be targeted through CB2 activation. CB2 has been studied as a promising therapeutic target. Future efforts should center around developing CB2 ligands that activate specific signaling pathways, as established in this paper, and determine which ones are effective in the inflammatory context of each pulmonary disease.

## Conclusion

Various studies suggest that monocyte/macrophage adoptive transplantation reverses inflammatory injury. However, these studies showed various signaling pathways, but the question is which signaling pathway is important among those to resolve the ALI/ARDS inflammation? Thus, the full therapeutic implications of CB2 agonists are still unknown. Determining the CB2 receptor agonist signaling pathway for reducing cytokine storm and inflammation by repolarizing inflammatory macrophages into reparative macrophages will have the greatest impact in a clinical context. Studies suggested that CB2 receptor agonists, lacking central unwanted side effects, may be promising therapeutic targets in lung inflammatory diseases by modulating the pulmonary immune system and converting inflammatory macrophages to the reparative stage.
